# Intramolecular Folding in Human ILPR Fragment with Three C-Rich Repeats

**DOI:** 10.1371/journal.pone.0039271

**Published:** 2012-06-25

**Authors:** Soma Dhakal, Javonne L. Lafontaine, Zhongbo Yu, Deepak Koirala, Hanbin Mao

**Affiliations:** Department of Chemistry and Biochemistry, Kent State University, Kent, Ohio, United States of America; University of South Florida College of Medicine, United States of America

## Abstract

Enrichment of four tandem repeats of guanine (G) rich and cytosine (C) rich sequences in functionally important regions of human genome forebodes the biological implications of four-stranded DNA structures, such as G-quadruplex and i-motif, that can form in these sequences. However, there have been few reports on the intramolecular formation of non-B DNA structures in less than four tandem repeats of G or C rich sequences. Here, using mechanical unfolding at the single-molecule level, electrophoretic mobility shift assay (EMSA), circular dichroism (CD), and ultraviolet (UV) spectroscopy, we report an intramolecularly folded non-B DNA structure in three tandem cytosine rich repeats, 5'-TGTC4ACAC4TGTC4ACA (ILPR-I3), in the human insulin linked polymorphic region (ILPR). The thermal denaturation analyses of the sequences with systematic C to T mutations have suggested that the structure is linchpinned by a stack of hemiprotonated cytosine pairs between two terminal C4 tracts. Mechanical unfolding and Br_2_ footprinting experiments on a mixture of the ILPR-I3 and a 5′-C4TGT fragment have further indicated that the structure serves as a building block for intermolecular i-motif formation. The existence of such a conformation under acidic or neutral pH complies with the strand-by-strand folding pathway of ILPR i-motif structures.

## Introduction

Tandem repeats of DNA residues are abundant throughout human genome [1]–[3]. Non-B DNA structures, such as G-quadruplex, i-motif, DNA cruciform, and H-DNA, can form in these tandem repeats [3]. Under physiological conditions, these non-B DNA structures can prevail over DNA duplex to regulate DNA processing or gene expression. From this perspective, the DNA sequence does not merely serve as a genetic code; it can also form a structure to interfere with replication or other biological processes [4]. During DNA replication for instance, it has been proposed that the presence of non-B DNA structures can cause deletion or addition of repeated sequences [5].

In human promoter regions, enrichment of cytosine and guanine repeats has been observed [1]. Four tandem repeats of cytosine and guanine rich sequences can host i-motif and G-quadruplex structures, respectively [6], [7]. The fact that these structures can form in promoter regions has led to the hypotheses that G-quadruplexes or i-motifs may regulate RNA transcriptions [8]. While various structures of G-quadruplex have been illustrated, much less information is available for the i-motif structure that can exist in the regions complementary to the G-quadruplex forming sequences [9]. I-motif is composed of a stack of hemiprotonated C:CH^+^ pairs [10]. Parallel orientation exists for C-rich repeats that form the C:CH^+^ pairing in both intermolecular [10], [11] and intramolecular [12]–[14] structures. The essential component of hemiprotonated C:CH^+^ pairs implies that acidic condition is necessary for the formation of this structure [15]. However, recent studies started to reveal that the structure can form even at physiological pH, [6], [16] especially under molecular crowding conditions [17] or with negative superhelicity of the DNA template [18]. The physiological significance of the i-motif structure is implied in recent discoveries that many proteins, such as single-stranded DNA binding proteins (SSB), helicases and other motor proteins, can specifically recognize C-rich sequences [19], [20]. In addition to Human genome, C-rich repeats are also found in Drosophila virilis DNA [21] and some cardiovirus RNAs [22]. These discoveries expose the i-motif to a broad range of host species for potential biological roles.

To function as a regulatory element *in vivo*, folding and unfolding of an i-motif structure are equally important [8]. It has been proposed that formation or dissolution of an i-motif undergoes either a strand-by-strand or a duplex-by-duplex pathway [8], [23], [24]. Observation of hitherto evasive intermediates helps to identify a specific pathway. However, due to the small quantity of these intermediates, they are highly difficult to investigate by *ensemble* techniques such as CD, NMR, X-ray crystallography. Single-molecule approaches offer unique capability to illustrate these structures. For example, our recent mechanical unfolding experiments on i-motif structures in the Insulin Linked Polymorphic Region (ILPR) has suggested that partially folded C-rich structures may adopt a triplex-like DNA structure employing three neighboring C-rich repeats among four available C-tracts [6]. Based on this, it is reasonable to assume that similar structures may also exist in a sequence with only three available cytosine-rich repeats. Compared to four or more C-rich tandem repeats, three neighboring C-rich repeats are expected to occur more frequently in human genome. This increases the opportunity for the C-rich structures to form *in vivo*, which sets a premise for the structures to play functional roles in biological processes. Since current algorithm only searches for regions that have more than three C-repeats for possible C-rich structures, formation of a stable structure in a sequence of three C-repeats will transform the searching algorithm. Recently, reports have shown a stable intramolecular structure in three tandem G-rich repeats [25], [26]. However, stand-alone structures in three tandem C-rich repeats have not been reported.

Here, we have used a DNA fragment with three C-rich tandem repeats, 5'-TGTC4ACAC4TGTC4ACA-3', derived from the ILPR region, to investigate the possible secondary structure formed in this sequence. We have observed a stable structure during mechanical unfolding experiments, CD and UV melting measurements, as well as native gel shift assays. The C:CH^+^ pair stacking in the structure has been suggested by pH titration during mechanical unfolding and CD measurements. The participating C4 tracts in the C:CH^+^ pairing have been identified with mutational analysis. The contour length and unfolding force measurement on the mechanical unfolding experiments have supported the parallel strand orientation in the folded structure. Based on these, we propose a possible structure linchpined by C:CH^+^ pair stacking. Furthermore, laser-tweezers and bromine footprinting experiments have shown that this structure can serve as a building block for i-motif structures. We anticipate the existence of a stable species in three tandem C-rich repeats not only adds a new possibility for gene regulation, but also generates a novel thought on the DNA based nanomaterials and biosensors.

## Results

### CD spectroscopy demonstrated that structures in the ILPR-I3 sequence contain hemiprotonated cytosine pair stackings

**Figure 1:**
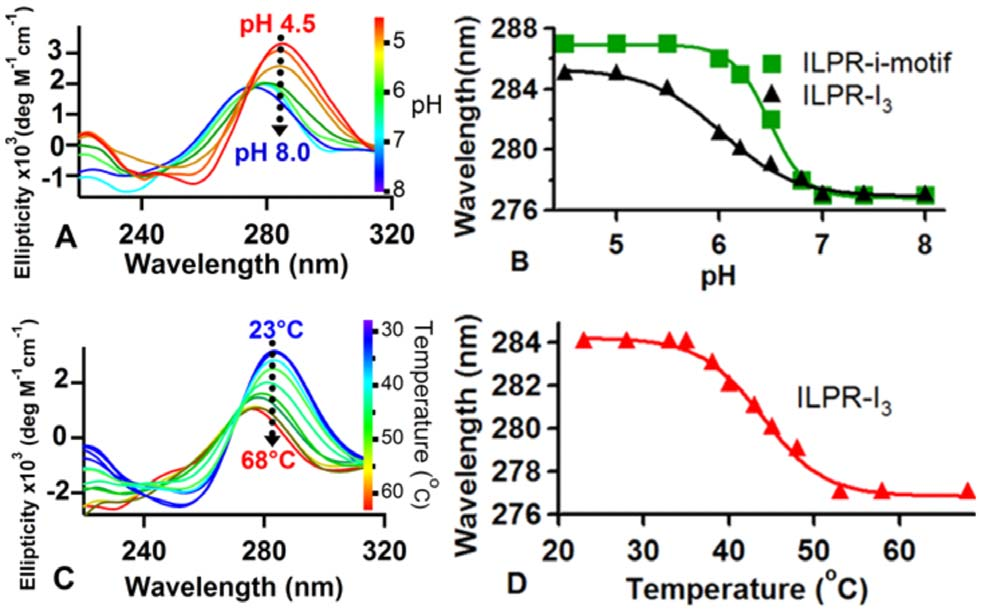
CD experiments of ILPR-I3 at different pH and temperature in a 10 mM sodium phosphate buffer with 100 mM KCl and 5 µM DNA concentration. (A) CD spectra of the ILPR-I3 in pH 4.5–8.0. (B) Peak wavelength *vs* pH for the ILPR-I3 (obtained from (A)) and ILPR-I4 (obtained from the CD spectra of the ILPR-I4 at pH 4.5–8.0, data not shown). (C) CD spectra acquired at 23–68°C (pH 5.5). (D) Peak wavelength *vs* temperature (obtained from (C)). The transition points in B) and D) are determined by sigmoidal fitting (solid curves).

First, we performed CD measurements to determine whether stable structures exist in the wild-type ILPR sequence, 5'-TGTC4ACAC4TGTC4ACA-3' (ILPR-I3, Table 1). The CD spectra at pH <6 showed a broad positive band at ∼285 nm and a negative band at ∼260 nm (Figure 1A). These CD signatures have been demonstrated previously for either intercalative [14] or non-intercalative C:CH^+^ pair stackings (see Discussion) [27]. When pH increased towards neutrality, the band evolved towards 277 nm, a signal characteristic of a random coil conformation [28]. This pH dependency is similar to those observed for the i-motif structure in the ILPR-I4 sequence, 5'-(TGTC4ACAC4)_2_TGT [6]. Figure 1B shows the direct comparison between these two species. Although both species demonstrated the sigmoidal dependency on pH, the structure in the ILPR-I3 showed a shallower transition compared to the i-motif formed in ILPR-I4. This observation reveals sluggish response of the ILPR-I3 to the pH, possibly due to less C:CH^+^ stacking involved in the structure. The decreased pH dependency of the structure in the ILPR-I3 is in agreement with that for a partially folded structure revealed by the single-molecule study on the ILPR-I4 sequence [6].

**Table 1:** Sequences of wild type ILPR-I4 and ILPR-I3, a scrambled sequence, and the mutants used in this study.

Oligonucleotides	Sequence
Wild Type (ILPR-I4)	5'-TGTCCCCACACCCCTGTCCCCACACCCCTGT-3'
Wild Type (ILPR-I3)	5'-TGTCCCCACACCCCTGTCCCCACA-3'
Scrambled Sequence(ILPR-S3)	5'-CCTCGCTCACACTCCGCTCACCAC-3'
Wild Type (ILPR-I1)	5'-CCCCTGT-3'
Mut-C4T	5'-TGT**T**CCCACACCCCTGTCCCCACA-3'
Mut-C5T	5'-TGTC**T**CCACACCCCTGTCCCCACA-3'
Mut-C6T	5'-TGTCC**T**CACACCCCTGTCCCCACA-3'
Mut-C7T	5'-TGTCCC**T**ACACCCCTGTCCCCACA-3'
Mut-C12T	5'-TGTCCCCACAC**T**CCTGTCCCCACA-3'
Mut-C11-14T	5'-TGTCCCCACA**TTTT**TGTCCCCACA-3'
Mut-C18T	5'-TGTCCCCACACCCCTGT**T**CCCACA-3'
Mut-C19T	5'-TGTCCCCACACCCCTGTC**T**CCACA-3'
Mut-C20T	5'-TGTCCCCACACCCCTGTCC**T**CACA-3'
Mut-C21T	5'-TGTCCCCACACCCCTGTCCC**T**ACA-3'

To provide further evidence that ILPR-I3 fragment folds into a secondary structure, we performed CD melting measurements. With increasing temperature, the 285 nm CD band became blue shifted to 277 nm, a signal indicative of non-structured DNA [29] (Figure 1C). In line with this observation, a melting transition temperature of 44±0.7°C was observed (Figure 1D). These CD measurements demonstrate that ILPR-I3 can fold into a thermally stable structure containing a stack of C:CH^+^ pairs.

### Gel shift assay and thermal denaturation analyses suggested that the folded structure is intramolecular

**Figure 2:**
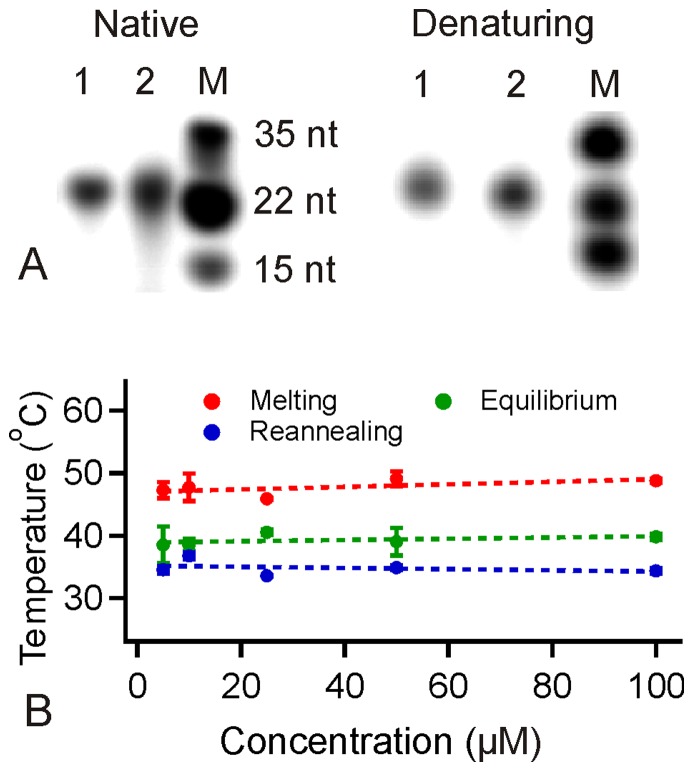
Electrophoretic mobility shift assays (EMSA) and thermal melting/reannealing of the ILPR-I3. (A) EMSA of the ILPR-S3 (scrambled DNA, lane 1) and the ILPR-I3 (lane 2) at 1 µM strand concentration. Lane 3 shows the DNA marker (M). Left panel, a native gel at pH 5.5. Right panel, a denatured gel (10% PAGE, 7×M urea). (B) Melting (*T*_1/2-*melting*_), reannealing (*T*_1/2-*reannealing*_), and equilibrium (*T*_m_) temperatures of the structure in the ILPR-I3 with 5 to 100 µM concentration. The melting and reannealing temperatures were determined by 295 nm UV-melting and UV-reannealing, respectively, in a 10 mM sodium phosphate buffer (pH 5.5) with 100 mM KCl. The equilibrium melting temperature (*T*_m_) of the ILPR-I3 was determined based on the non-equilibrated melting and reannealing curves (see Materials and Methods).

To determine whether the ILPR-I3 folds intramolecularly, we performed thermal denaturation (UV) and gel shift assays. Due to compact conformations, intramolecularly folded DNA structures are expected to migrate faster in gel electrophoresis than unstructured DNA of the same length. Indeed, we observed that a fraction of the ILPR-I3 showed faster electrophoretic mobility (notice the front tail in lane 2, Figure 2A, left panel) compared to an unstructured ILPR-S3 sequence (lane 1, Figure 2A, left panel, see Table 1 for the sequence) in the native electrophoretic mobility shift assay (EMSA) at pH 5.5. To ensure that the front tail on the ILPR-I3 (lane 2 of the native gel in Figure 2A) was not due to artifacts, three native gel experiments were performed (see Figure S1 for the gel image of another experiment). In all three experiments, the front tail was consistently observed in the ILPR-I3, but it was not shown in the scrambled sequence, neither was it observed in the denaturing gel.

The unstructured ILPR-S3 was confirmed by the 277 nm CD band (Figure S2) and lack of UV melting transition (data not shown). In addition, the smeared band [30] observed for ILPR-I3 (Lane 2, Figure 2A, left panel) under native condition suggests that a fraction of ILPR-I3 was folded intramolecularly. As a control, both ILPR-I3 and ILPR-S3 showed similar electrophoretic mobilities in a denatured PAGE gel (Figure 2A, right panel). These results suggest that an intramolecularly folded structure forms in the ILPR-I3 fragment.

Previous investigations have shown that, in contrast to intermolecular DNA structures, *T*_m_ of an intramolecular structure is independent of DNA concentrations [29], [31]. We measured the melting (*T*_1/2-melting_), reannealing (*T*_1/2-reannealing_), and equilibrium (*T*_m_) temperatures according to the procedures described in literature [32]–[34] while varying the concentration of the ILPR-I3 from 5 to 100 µM at pH 5.5 (Figure 2B). As shown in Figure 2B, we found that *T*_1/2-melting_, *T*_1/2-reannealing_ and equilibrium melting temperature (*T*_m_) remained unchanged over a 20-fold variation in strand concentration, confirming the intramolecular nature of the structure in the ILPR-I3.

### Single-molecule study provided the direct evidence for intramolecular folding of the ILPR-I3 from acidic to neutral pH

**Figure 3:**
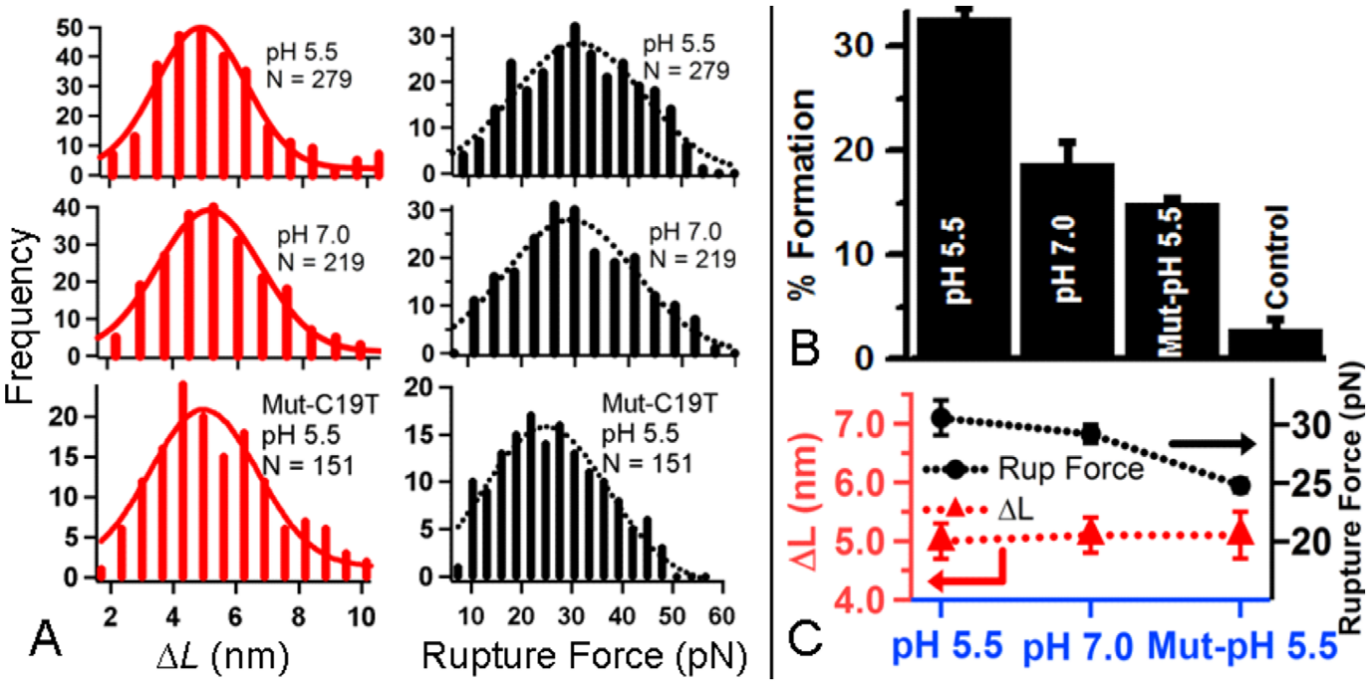
Single-molecule study of the ILPR-I3 at different pH using laser tweezers. (A) Change in contour length (Δ*L*) (left panel) and rupture force histograms (right panel) of the ILPR-I3 and the Mut-C19T at different pH (23°C). The histograms are fitted with Gaussians (solid and dotted curves). *N* depicts the number of experiments. (B) Percentage formation of the ILPR-I3 at pH 5.5 and 7.0 (“pH 5.5″ and “pH 7.0″), the Mut-C19T at pH 5.5 (“Mut-pH 5.5″), and a DNA construct that does not contain the C-rich sequence at 23°C (“Control”). (C) Comparison of Δ*L* (triangles linked by the red dotted line at the bottom) and rupture force (filled circles linked by the black dotted line at the top) between the ILPR-I3 and the Mut-C19T fragments. Notations are the same as described in (B).

The decisive evidence for the intramolecular nature of the structure in the ILPR-I3 came from mechanical unfolding studies using laser tweezers. To this end, the ILPR-I3 fragment was sandwiched between two dsDNA handles. One of the handles was labeled with biotin and the other with digoxygenin (Dig) at the free end. This setup allowed the DNA construct to be tethered between the two beads functionalized with streptavidin and anti-Dig antibody, respectively (see Materials and Methods and Figure S3 for details). The native flanking sequences, TGT and ACA, were incorporated at the two ends of the ILPR-I3 to reduce the steric hindrance between the folded structure and the dsDNA handles. To unfold the possible secondary structure in the ILPR-I3, the DNA construct tethered between the two optically trapped beads was stretched by moving the two beads apart. In the ensuing force-extension (F-X) curves, we observed a sudden drop in the force, which indicated the unfolding of a DNA secondary structure (Figure S3). Since the experiment was performed on individual molecules, the rupture event must represent the unfolding of an intramolecular structure, whose size is quantified by the change in contour length (Δ*L*, Figure 3A, left panel). The average unfolding forces for these species were 31±1 pN at pH 5.5 and 30±1 pN at pH 7.0 (Figure 3A, right panel), demonstrating that folded structures were mechanically robust.

It is expected that the structure employing hemiprotonated cytosine pair stacking is favored under acidic conditions. When we analyzed the percentage formation at pH 5.5 and 7.0 (see Materials and Methods), indeed, we observed a decreased percentage formation at pH 7.0 (19±2%) compared to pH 5.5 (33±1%) (Figure 3B). In comparison, a control DNA construct containing only the double-stranded DNA handles (see Materials and Methods) demonstrated negligible unfolding events of 3±1% (3 out of 105 curves). The substantial formation of the structure at neutrality confirmed the pH titration results that the ILPR-I3 structure was less pH dependent compared to the i-motif (Figure 1B). This fact adds new evidence that C-rich structures containing C:CH^+^ pairs can form at pH 7.0, [18], [24], [35] which sets a premise for biological roles of these structures.

To illustrate the structure that employs C:CH^+^ pair stacking, we evaluated four most probable candidates in Figure S4. First, we determined the end-to-end distance (*x*) of each structure from coordinates of similar PDB structures [11], [36]. This value was then used to derive Δ*L* (change in contour length) from the equation, *L* = Δ*L* + *x*, [6] where the contour length of each structure was calculated by *L* = *N × L*_single nucleotide_, (*N* is the number of nucleotides in each structure and *L*_single nucleotide_ = 0.43 nm [37]–[39], see Supporting Information for details). Among the four species shown in Figure S4, only the structure utilizing the first and the third C4 tracts in a parallel orientation for the C:CH^+^ pair stacking yields a Δ*L* of 5.3–5.9 nm, which falls into the observed Δ*L* of 5.2±0.4 nm at pH 7.0 and close to the Δ*L* of 5.0±0.1 nm at pH 5.5 (Figure 3A, left panel). Such a strand orientation is consistent with the finding that only parallel arrangement is possible for the C:CH^+^ pair stacking, which requires *anti* glycosidic bonds in cytosines to avoid steric effects [40].

If the structure does employ the first and the third C4 tracts with parallel orientation, then the mutation of any cytosine residues in these two C4 tracts is expected to destabilize the structure. To test this, we prepared a DNA construct in which the second cytosine (C19) in the third C4 tract was mutated to T (Mut-C19T in Table 1, 5'-TGTC4ACAC4TGTC**T**CCACA). After we mechanically unfolded the mutant at pH 5.5, we found Δ*L* of the mutant (5.1±0.4 nm) was identical with that of the wild type; whereas the rupture force (24.8±0.6 pN) was significantly lower (Figure 3 A&C). The former observation indicates that the C19T mutation still allows the folding of a secondary structure, which is consistent with the previous finding that intercalative C:CH^+^ pairs are not necessary for a folded structure [35]. The latter observation clearly shows that the structure has weakened stability, which is likely due to the loss of one stack of C:CH^+^ pair as a result of the C19T mutation. Compared to the structure in the wild-type ILPR-I3 at pH 5.5, the percentage formation of the structure in the C19T DNA (15±1%) is decreased by ∼18% (Figure 3B). This is consistent with the less stable structure due to reduced C:CH^+^ pair stacking in the mutant.

### Thermal stability analysis and CD measurements of the mutants suggested cytosine pair stacking between distally located C4 tracts

**Figure 4:**
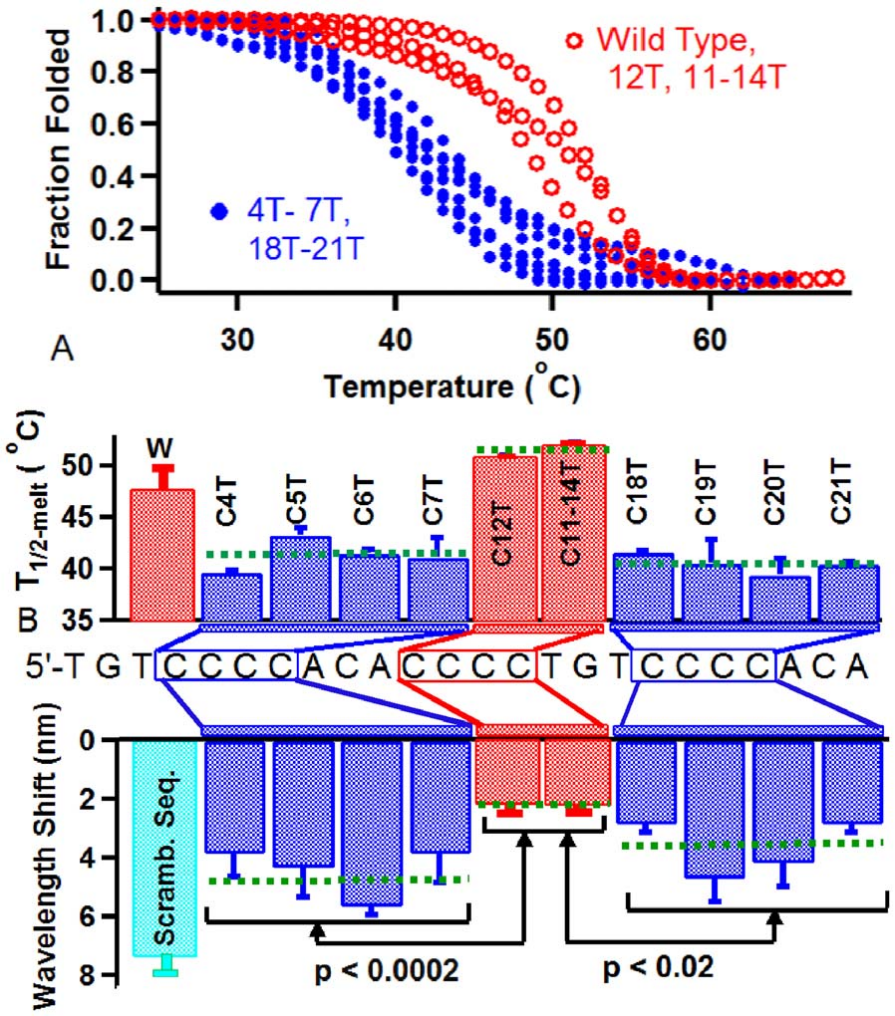
Mutation analysis in a 10 mM sodium phosphate buffer (pH 5.5) with 100 mM KCl. (A) 295 nm UV melting curves of the ILPR-I3 (“Wild Type”) and the mutants at 10 µM concentration. (B) Top panel, *T*_1/2-melt_ of the mutants and the ILPR-I3. “W” depicts the wild type ILPR-I3. Bottom panel, CD peak shift of the mutants and the scrambled sequence (ILPR-S3) with respect to the 285 nm peak in the ILPR-I3. The horizontal dotted lines (green) represent the average value for each C4 tract. Statistical treatment is represented by the *P* values in the bottom panel. Please refer to Table 1 for DNA sequences.

To further confirm the specific C4 tracts involved in the C:CH^+^ stacking, we systematically mutated cytosines to thymines in each C4 tract of the ILPR-I3 and evaluated the thermal stability of these mutants (Table 1). When a particular cytosine in the C:CH^+^ pairing is mutated to thymine, *T*_1/2-melting_ is expected to be lower than that of the wild type [29]. Figure 4A shows the results of the thermal stability analysis monitored at 295 nm on 10 µM mutants at pH 5.5. Two populations are strikingly clear in Figure 4A. One is clustered around the wild-type ILPR-I3 with similar melting temperatures; while the other with 3–10°C lower in *T*_1/2-melting_ is located close to the Mut-C19T, a mutant with compromised stability demonstrated by the single-molecule experiments. A close analysis reveals that the mutations involving the second C4 tract lead to the population with *T*_1/2-melting_ comparable to that of the wild-type ILPR-I3; whereas the mutations on the first and the third C4 tracts result in populations with lowered *T*_1/2-melting_. Such a distribution clearly indicates that the first and the third C4 tracts participate in the stacking of hemiprotonated cytosine pairs.

This conclusion was fully supported by structural analysis on all mutants using CD measurements (Figure S2). Compared to the wild-type ILPR-I3 whose 285 nm CD band is indicative of the C:CH^+^ pair stacking (either intercalative or non-intercalative, see Discussion), all mutants show blue shifted bands in Figures 4B and S2. In fact, the DNA with a scrambled sequence, ILPR-S3, shows such a complete shift that a broad band centered at 277 nm is observed. This indicates an unstructured conformation for this sequence, a result consistent with the EMSA observation (Figure 2A). The mutants involving the first and the third C4 tracts have significantly larger blue shifts than those involving the second C4 tract (p<0.0002 for the first C4 tract and p<0.02 for the third C4 tract). Since the blue shift from 285 nm to 277 nm suggests the switching of the C:CH^+^ stacking to an unstructured conformation, these results well explain the thermal analysis data from a structural perspective.

**Figure 5:**
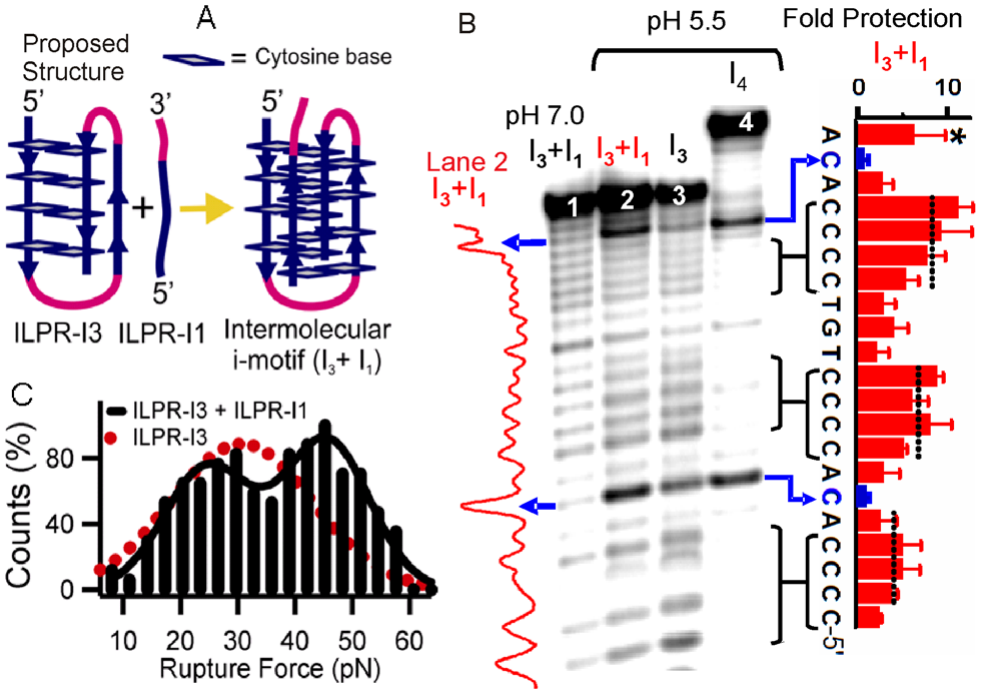
Formation of an intermolecular i-motif. (A) Schematic of the formation of an intermolecular i-motif. The proposed structure in the ILPR-I3 is shown on the left. Each C:CH^+^ pair is represented by two opposite rectangles. (B) PAGE gel image of the Br_2_ footprinting experiment. Lane 1, the ILPR-I3/ILPR-I1 (I_3_+I_1_) mixture at pH 7.0. Lane 2, the I_3_+I_1_ sample at pH 5.5. Lane 3, the ILPR-I3 (I_3_) at pH 5.5. Lane 4, the ILPR-I4 (I_4_) at pH 5.5. The band intensity for lane 2 is shown to the left of the gel. The fold protection for the I_3_+I_1_ sample at pH 5.5 is shown to the right. The dotted vertical lines indicate the average fold protection for each C4 tract. The blue arrows indicate the loop cytosines. Error bar represents the standard deviation of three independent experiments. The blue arrows indicate the cytosines in the ACA section of each fragment. Note that the fold protection for adenines at 3'-end (indicated by asterisk *) is not accurate since they are close to the uncut oligo. (C) Normalized rupture force histogram for the I_3_+I_1_ sample at pH 5.5. The solid black curve represents a two-peak Gaussian function. The dotted curve is the Gaussian fit for the rupture force histogram of the ILPR-I3 at pH 5.5.

The thermal stability experiments and CD analysis described here suggest that the structure in the ILPR-I3 is joined by hemiprotonated cytosine pair stacking utilizing the first and the last C4 tracts in the sequence. Such a conformation (Figure 5A) is also consistent with the proposed structure from single-molecule investigations.

### The intramolecularly folded ILPR-I3 served as a building block for intermolecular i-motif structures

We anticipate that in the presence of the ILPR-I1 fragment, 5′-CCCCTGT, folding of the ILPR-I3 can evolve into an intermolecular i-motif (Figure 5A). Bromine footprinting experiments provided direct support to this expectation. It has been shown that cytosines in the C:CH^+^ pair stacking are more protected against Br_2_ than those in the single stranded region [41]. At pH 5.5, the Br_2_ footprinting on the ILPR-I3/ILPR-I1 mixture (1∶1 molar ratio) showed more protection of each C4 tract in the ILPR-I3 fragment compared to that at pH 7.0 (Figure 5B, lanes 1&2 and the fold protection shown to the right). This result can be well explained by the formation of an intermolecular i-motif between the two oligos at pH 5.5, but not at pH 7.0. In accordance with the i-motif formation at pH 5.5, C4 tracts are more protected than the cytosines in the ACA sections, which remain single-stranded (lane 2 and the fold protection pattern). Figure S5 shows that fold protections of the corresponding C4 tracts in the ILPR-I3/ILPR-I1 mixture and the ILPR-I4 fragment are similar. Since ILPR-I4 is known to form an i-motif at pH 5.5 [6], this result again supports the formation of an intermolecular i-motif between the ILPR-I3 and the ILPR-I1. It is noteworthy that the C4 tracts in the ILPR-I3 sequence (Figure 5B, lane 3 and Figure S5) show much less protection against Br_2_ compared to either the ILPR-I4 sequence or the ILPR-I3/ILPR-I1 mixture. This may reflect the fact that the C4 tracts of the structure in the ILPR-I3 have less steric hindrance compared to those in i-motifs.

The formation of an intermolecular i-motif was further supported by mechanical unfolding experiments. The rupture force histogram showed two populations when the ILPR-I3 was unfolded in the presence of 10 µM ILPR-I1 (Figure 5C). Based on the close values of the rupture force between the two (compare the red and the left black population in Figure 5C), the population with 24±2 pN rupture force was assigned to the intramolecular ILPR-I3 structure. The population with increased rupture force of 45±1 pN was likely an intermolecular i-motif. This assignment is based on the fact that structures of intermolecular nature should have smaller unfolding rate constant (*k*_unfold_) compared to that of intramolecular structures [42]. Indeed, the unfolding rate constant for the 45 pN species, *k*_unfold_ = 7.2×10^−5 ^s^−1^, is much smaller than that for an intramolecular i-motif, *k*_unfold,intramolecular_ = 3.7×10^−3 ^s^−1^ (Figure S6). Taken together, the mechanical unfolding and footprinting results clearly indicate that the ILPR-I3 structure can serve as a building block for intermolecular i-motif. To the best of our knowledge, the unfolding experiments shown here represent for the first time an intermolecular i-motif has been investigated at the single-molecule level.

## Discussion

CD has been extensively used to characterize the i-motif structures in the C-rich oligos. The CD spectrum with a positive peak at ∼280–288 nm and a negative trough near 260 nm indicates the formation of i-motif structures. Not only intercalative C:CH^+^ stackings in a typical i-motif structure show these CD features, similar CD spectrum has also been observed for the non-intercalative C:CH^+^ stacking in duplex DNA [27]. The latter observation has broadened the scope of CD to characterize the structures involving non-intercalative C:CH^+^ pair stacking. Our study exploits this capability to characterize secondary structures in the ILPR-I3. Using the DNA concentration (5 µM) that favors the intramolecular folding (Figure 2B), we observed characteristic CD peaks for the C:CH^+^ stacking (the positive peak near 285 nm and the negative trough near 260 nm in Figures 1A and 1C). The pH dependency of the 285 nm peak (peak gradually blue shifted with increasing pH (4.5 to 8.0), see Figures 1A and 1C) further supported the presence of the pH sensitive C:CH^+^ stacking in the ILPR-I3.

It is possible that the ILPR-I3 structure may assume either a parallel or antiparallel strand arrangement (Figure S4). With the antiparallel orientation, structures resemble a hairpin in which the stem is composed of the C:CH^+^ pairs (Figure S4 C-D). Thus, the unfolding geometry used in current laser-tweezers experiments is equivalent to unzipping a hairpin, which requires around 15 pN [43]. However, the rupture forces observed here, 31 and 30 pN at pH 5.5 and pH 7.0 (Figure 3A, right panel), respectively, are significantly larger than this value. Instead, they are within the force range required to “slide” open a duplex DNA, [44] which shares the same geometry as the unfolding of the structures with parallel strand orientations (Figure S4 A–B).

Compared to the *ensemble* average measurements such as NMR and CD, the laser-tweezers based single-molecule method is highly sensitive. For example, at pH 7.0, laser tweezers revealed that 19% of the population is folded in the ILPR-I3 (Figure 3B). However, under the same condition, the 285 nm CD signal characteristic of the C:CH^+^ stacking was masked by the broad band at 277 nm, which is the signature from the main, unfolded population (Figure 1A). Therefore, although our laser-tweezers method does not produce structural information at atomic details as revealed by NMR or X-ray measurements, its highly sensitive nature has enabled it to probe the structures for species with minute quantity.

Apart from the sensitivity, laser tweezers have a unique capability to measure the mechanical stability of DNA structures [6], [7], [45]–[47]. Motor proteins, such as DNA/RNA polymerases and helicase, generate a load force during their enzymatic cycles [48]–[51]. Recent finding has revealed that DNA G-quadruplex and i-motif have mechanical stabilities [6], [7] comparable to the stall force of polymerases, suggesting they may play significant roles to regulate polymerases from the mechanical perspective alone. Here, the C-rich structure in the ILPR-I3 (*F*_unfold_ = 31 pN) shows a similar mechanical stability, suggesting a similar capability compared to other non-B DNA structures. That the ILPR-I3/ILPR-I1 mixture can form an intermolecular i-motif implies that the ILPR-I3 structure serves as an important building block *en route* to the i-motif folding. Previously, 3+1 G-quadruplex assemblies (three G-tracts from one strand and one G-tract from another strand) have been reported by laser tweezers, NMR, and AFM studies [26], [52], [53]. These results provide evidence that folding or unfolding of DNA tetraplexes may follow a strand-by-strand, [23] instead of duplex-by-duplex pathway [10], [24].

In summary, we have shown the existence of a stable structure in the ILPR sequence with three C-rich repeats. Our results show that folded species in the ILPR-I3 is mechanically and thermodynamically stable. The structure is stabilized by the first and the third C4 tracts *via* hemiprotonated cytosine pair stacking with a parallel strand arrangement.

## Materials and Methods

### Materials

All oligonucleotides were purchased from Integrated DNA technologies (IDT, Coralville, IA) and purified by 10% denaturing PAGE. Other than those specifically labeled, all chemicals were purchased from VWR.

### CD Spectroscopy

PAGE purified oligonucleotides were used to prepare 5 µM solutions (200 µL) in 10 mM sodium phosphate buffer with 100 mM KCl at a given pH. DNA samples were heated to 95°C for 10 minutes and transferred to an ice-water bath before acquiring CD spectra. The CD spectra were collected in a quartz cuvette with a 1 mm optical path length at a given temperature or pH using a Jasco-810 spectropolarimeter (Easton, MD). For the experiments performed above room temperature, mineral oil was added on top of the solution to prevent evaporation. The spectra were averages of three scans acquired over the wavelength range of 220–320 nm at a scan rate of 50 nm/min. To avoid the background signal from buffers, the spectra were baseline-corrected and smoothed using Savitzky-Golay function.

### UV Spectroscopy

The basic procedure of sample preparation in UV experiments was the same as the CD experiment described above. All UV experiments were performed in 10 mM sodium phosphate buffer with 100 mM KCl at pH 5.5. In the thermal analysis of the ILPR-I3, UV measurements were performed over the concentration range of 5 to 100 µM. In the thermal analysis of the mutants, 10 µM DNA was used. The UV-melting experiments were performed at 295 nm with a heating rate of 0.5°C/min in a quartz cuvette (1 cm optical path length) using a Varian Cary 300 spectrophotometer. To correct for buffer signals, the absorbance of the buffer-only solution was subtracted from the UV melting curves. All melting curves were baseline corrected, normalized and plotted as the fraction folded *vs* temperature and the transition temperatures (*T*_1/2_) were calculated for melting and reannealing processes as described elsewhere [32], [33]. The equilibrium melting temperature (*T*_m_) of the ILPR-I3 was determined based on the non-equilibrated melting and reannealing curves according to the procedures described in the literature [32], [34].

### Electrophoretic Mobility Shift Assay (EMSA)

The ILPR-I3, the scrambled sequence ILPR-S3, and the Mut-C19T (see Table 1 for sequences) were 5'-end labeled with [γ-^32^P] ATP (Perkin Elmer, Waltham, MA) by incubating the DNA with T4 polynucleotide kinase (T4 PNK, New England Biolab, NEB, Ipswich, MA) for 1 hr at 37°C followed by heat inactivation of the T4 PNK for 10 min at 70°C. The radiolabeled oligonucleotides were then purified by MicroSpin™ G-25 columns (GE Healthcare, Buckinghamshire, UK).

These radiolabeled oligos (1 µM final concentration) were prepared in a 10 mM sodium phosphate buffer (pH 5.5) with 100 mM KCl. The oligo samples were heated to 95°C for 10 min and transferred to an ice-water bath for fast cooling and analyzed in 10% native PAGE gels. The sodium phosphate running buffer with 100 mM KCl was changed with fresh buffer every 45 min to maintain the salt concentration during the electrophoresis. A control experiment was performed under the denaturing condition (7 M urea, 10% denaturing PAGE).

### DNA Constructs

Detailed description on the preparation of DNA constructs has been described elsewhere [6]. In our DNA construct that comprised of three fragments, a 24-mer ILPR-I3 sequence was sandwiched between two dsDNA handles. One of the dsDNA handles (2028 bp) was labeled with biotin at the 5' end using PCR amplification of the pBR322 plasmid (NEB) and a biotin labeled primer (IDT). Another dsDNA handle (2690 bp) was obtained by sequential digestion of the pEGFP plasmid (Clontech, Mountain View, CA) with the SacI and the EagI (NEB) restriction enzymes. The fragments were gel purified and labeled with digoxygenin (Dig) at the 3' end using 18 µM dig-dUTP (Roche, Indianapolis, IN) and terminal transferase (Fermentas, Glen Burnie, MD).

The sequence containing the ILPR-I3 (bold, underlined), 5'-*CTA GAC GGT GTG AAA TAC CGC ACA GAT GCG* **TGT CCCC ACA CCCC TGT CCCC ACA** *GCC AGC AAG A* *CG TAG CCC AGC GCG TC*, was phosphorylated at the 5' end using the T4 PNK (NEB) and mixed with two other oligonucleotides, 5'-CGC ATC TGT GCG GTA TTT CAC ACC GT and 5'-GGC CGA CGC GCT GGG CTA CGT CTT GCT GGC, which were complementary to the flanking regions (italic) of the sequence given above. The mixture of the oligos was heated to 97°C for 5 min and annealed slowly to room temperature so that the ILPR-I3 containing ssDNA was sandwiched between the flanking dsDNA. The annealed DNA fragment was ligated with the biotin-labeled 2028 bp dsDNA handle on the one end and the Dig-labeled 2690 bp dsDNA on the other using T4 DNA ligase (NEB). The ligated DNA construct was purified by a Centricon (50,000 MWCO, Millipore, Bedford, MA). The same procedure was used to prepare the DNA construct containing the Mut-C19T fragment (see Table 1 for the sequence) and the control construct in which the ILPR-I3 sequence was deleted while the dsDNA handles remained intact.

### Anti-Dig-Antibody Coated Beads

Two milliliters of protein G polystyrene particles (2.10 µm diameter, Spherotech, Illinois,) were spun down at 3000 rpm for 3 min and the pellet was re-suspended in 1 mL of a crosslinking buffer made of 100 mM each of Na_2_HPO_4_ and NaCl (pH 8.5). To this solution, 60 µL (1 µg/mL in 100 mM sodium phosphate buffer, pH 7.4) of polyclonal anti-Dig-antibody (Roche, Indianapolis, IN) and 30 µL of freshly dissolved dimethyl pimelidate dihydrochloride (DMP, Pierce Protein Research Products, 50 mg/mL in the crosslinking buffer) were added and tumbled for 1 hr at room temperature. The reaction was terminated by adding a 50 mM Tris buffer (pH 8.0). The beads were spun down and re-suspended in a 100 mM phosphate buffer (140 mM NaCl, 0.02% NaN_3_, pH 7.0).

### Single-Molecule Experiments

The house-made laser tweezers instrument used for single molecule experiments has been reported elsewhere [54], [55]. To start single-molecule experiments, the end-labeled DNA construct was incubated with anti-Dig-coated polystyrene particles (2.10 µm diameter) for 1 hr to allow the DNA molecules to bind to the bead surface *via* the Dig/anti-Dig antibody linkage. The anti-Dig coated beads carrying the DNA molecules and the streptavidin coated beads (0.97 µm diameter, Spherotech) were dispersed in 700 µL of 10 mM sodium phosphate buffer with 100 mM KCl at pH 5.5 or 7.0. They were separately injected into a reaction chamber. The two types of beads were separately trapped by two laser foci in the reaction chamber. The bead carrying the DNA molecules on its surface was moved towards the streptavidin coated bead so that the free end of the attached DNA molecule could bind to the latter bead *via* the biotin/streptavidin linkage. Once the DNA was tethered between the beads, the anti-Dig coated bead was moved away at a loading rate of ∼5.5 pN/s until the secondary structure in the DNA construct was unfolded during the extension. After reaching to a specific force, the force was relaxed to zero at the same loading rate to allow the structure to refold before subsequent pulling. The single tether was confirmed by a single breakage event for the tethered DNA construct. All data were recorded in Labview™ and analyzed with Matlab™ and Igor™ programs. The rupture force was measured directly from the force-extension (F-X) curves; whereas the change in contour length (Δ*L*) was calculated from the two data points flanking the rupture event using the worm like chain (WLC) model given below (eqn 1):
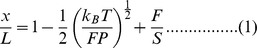
where *x* is the end-to-end distance, *k*_B_ is the Boltzmann constant, *T* is absolute temperature, *P* is the persistent length (51.95 nm [56]), *F* is the force, and *S* is the elastic stretch modulus (1226 pN [56]).

### Calculation of Percentage Formation

Percentage formation of secondary structures in DNA fragments at pH 5.5 or 7.0 was calculated as the ratio of the DNA tethers that contain folded secondary structures *vs* the total number of DNA tethers at a given pH. The DNA tether was counted only once to avoid repetitive counting.

### Br_2_ Footprinting

The detailed procedure for bromine footprinting experiment has been described elsewhere [6]. Briefly, the ILPR-I4 and ILPR-I3 sequences (see Table 1) were end-labeled with [*γ*-^32^P] and purified as described above (see EMSA, Materials and Methods). The labeled oligos were prepared in a 10 mM sodium phosphate buffer (pH 5.5 or 7.0) supplemented with 100 mM KCl and 1 µM unlabeled oligos in a 50 µL reaction volume. In the intermolecular i-motif experiments, 1**µM ILPR-I1 was added to the ILPR-I3 prior to the incubation. The samples were heated to 95°C for 5 min followed by slow cooling to room temperature in ∼3 hr. Cytosine-specific cleavage was performed to probe the cytosine residues by incubating the DNA samples with molecular bromine generated *in situ* from the reaction between KBr (1 µL, 20 mM ) and KHSO_5_ (1 µL, 10 mM ) [41]. The reactions were performed for 3 min at room temperature, terminated by adding a stop buffer (1 mg/mL sheared salmon sperm DNA, 300 mM CH_3_COONa, and 4 mM HEPES) followed by ethanol precipitation. The DNA pellet was re-suspended in 70 µL of 10% piperidine and incubated at 95°C for 30 min. It was dried by vacuum centrifuge and the resulting DNA fragments were resolved on a 10% denaturing PAGE gel. The gel was dried, exposed to a phosphorimager screen for overnight (∼12 hr), and scanned with a Typhoon 8600 instrument (GE Healthcare). The Kodak Digital Camera Software (Eastman Kodak Company, Rochester, NY) was used to measure the band intensity. The intensity of each band for a particular sample at pH 5.5 or 7.0 was quantified separately, corrected for the background, and normalized with the band intensity of the cytosine in the ACA section (C9 from the 5′ end). Fold protection was calculated as the ratio of the band intensity at pH 7.0 to that of the corresponding band at pH 5.5.

## Supporting Information

Figure S1 Electrophoretic mobility shift assays (EMSA) of the ILPR-I3. EMSA of the ILPR-S3 (scrambled DNA, lane 1) and the ILPR-I3 (lane 2) at 1 µM strand concentration. Lane 3 is the DNA marker (M). Left panel, a native gel at pH 5.5. Right panel, a denatured gel (10% PAGE, 7 M urea).
(DOC)

Figure S2 CD spectra of sequences described in Table 1. CD spectra of the mutants with mutation sites in each of the three C4 tracts in the ILPR-I3 are plotted in A), B), and C), respectively. The spectra of ILPR-I3 (red) and the scrambled sequence (black) are also included in each Figure for direct comparison. These CD experiments were performed at 5 µM oligonucleotide concentration in a 10 mM sodium phosphate buffer (pH 5.5) with 100 mM KCl at 23°C.
(DOC)

Figure S3 A typical force-extension (F-X) curve obtained from the mechanical unfolding of the secondary structure in the ILPR-I3 (5'-TGT CCCC ACA CCCC TGT CCCC ACA) at pH 5.5. The unfolding event (∼5 nm) is highlighted by a dashed green circle. Black curve is the WLC fitting of the relaxing curve. Inset is the schematic of the laser tweezers experiment.
(DOC)

Figure S4 Four possible structures that employ C:CH^+^ pair stacking in the ILPR-I3 sequence. The three C4 tracts are shown in blue and other regions are shown in red for structures in (A)–(D). The unfolding direction for each structure is shown by black arrows labeled with “F”. Notice the structures with free C4 tracts at the 5'-end yield Δ*L* values identical to those of B and D, and therefore, they are not shown here. The change in contour length for each structure, Δ*L*, was calculated (see Table S1 for Δ*L* values) using the equation, Δ*L = N* ×*L*_single nucleotide_ – *x* (S1), where *N* is the number of nucleotides involved in the secondary structure, *L*_single nucleotide_ is the contour length for each nucleotide, and *x* is the effective end-to-end distance for the folded structure. The *x* remains the same for the structures A and B, as well as the structures C and D for the mechanical unfolding experiments. To calculate Δ*L*, the contour length for single nucleotide, *L*_single nucleotide_ = 0.43 nm, was used as reported (References S1 1–3). The *x* for structures C&D is 1.5 nm, which is the average inter-phosphate distance obtained from the literature (References S1 4–5). The *x* for structures A&B was estimated as the hypotenuse (see the green triangles in the top panel) to the rise of the four stacking C:CH^+^ pairs (the opposite side) and the inter-phosphate distance between the two C4 strands (1.5 nm, the adjacent side). Since the C:CH^+^ stacking resembles double stranded DNA (dsDNA), we set the lower limit of the rise per C:CH^+^ as 0.34 nm (single base pair rise in dsDNA) (References S1 6). The upper limit of the rise per C:CH^+^ is set at 0.66 nm, which is the average rise between the two intercalative C:CH^+^ stacking pairs determined from the known i-motif structures (PDB Codes; 1YBL, 1G22, 1EL2 and 1CNO) (References S1 7–10). This calculation yielded the rise of the four C:CH^+^ pairing between 1.0 and 2.0 nm (shown in the left triangle) for structures A and B. Based on this, the *x* was calculated as 1.8–2.5 nm for these two structures. Using eqn S1, these values yielded Δ*L* of 5.3–5.9 nm and 2.3–2.9 nm for structures A and B, respectively (summarized in the Table S1). As shown in Table S1, the observed Δ*L* (5.0±0.1 nm at pH 5.5 and 5.2±0.4 nm at pH 7.0) matched with the expected range of Δ*L* (5.3–5.9 nm) for structure A only.
(DOC)

Figure S5 Intensity scan for ILPR-I3 bands (the green trace to the left of the gel) and the fold protection for ILPR-I4 (I_4_, black) and ILPR-I3 (I_3_, green) for Br_2_ footprinting in a 10 mM sodium phosphate buffer at pH 5.5 with 100 mM KCl. Note that this gel is identical with that in Figure 5B (see Materials and Methods for fold protection calculation). Samples in different lanes are labeled according to Figure 5B. The cytosines in ACA sections are indicated by blue arrows in the intensity scan and blue bars in the fold protection graphs. The C4 tracts in the gel are highlighted with corresponding sequences. Error bars represent the standard deviations calculated from three independent experiments.
(DOC)

Figure S6Calculation of the unfolding rate constant (*k*_unfold_) at 0 pN for the intramolecular i-motif (“ILPR-I4”, calculation based on published data (References S1 References S1 11)) and the 45 pN population in the ILPR-I3/ILPR-I1 mixture (“ILPR-I3+ILPR-I1”) from the plot of ln[*r* ln*(1/N)*] versus rupture force. We used the equation S2 to estimate the *k*_unfold_, (References S1 12)_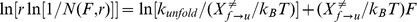 (S2)_ where *r* is the loading rate (5.5 pN/s), *N (F,r)* is the fraction of folded molecules at force *F* and loading rate *r*, and 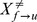 is the distance from the folded state to the transition state along the unfolding coordinate. *k*_unfold_ is obtained from the linear fit (solid black lines) in the graph. This calculation yielded *k*_unfold_ of 3.7×10^−3 ^s^−1^ and 7.2×10^−5 ^s^−1^, respectively, for the intramolecular ILPR i-motif and the 45 pN population in the ILPR-I3/ILPR-I1 mixture. Notice a randomized deconvolution of the two populations (24 and 45 pN) in the ILPR-I3/ILPR-I1 mixture (Figure 5C, black histogram) was used (References S1 11).(DOC)

Table S1 Calculation of contour length change (Δ*L*) of the four possible candidates shown in Figure S4.
(DOC)

References S1 Supporting Information References.
(DOC)
