# Supplementary material for: Intramolecular Folding in Human ILPR Fragment with Three C-Rich Repeats
Source: PLoS One. 2012 Jun 25;7(6):e39271. doi: 10.1371/journal.pone.0039271 (PMC3382603; doi:10.1371/journal.pone.0039271)
Supplement: Figure S1 — Electrophoretic mobility shift assays (EMSA) of the ILPR-I3. EMSA of the ILPR-S3 (scrambled DNA, lane 1) and the ILPR-I3 (lane 2) at 1 µM strand concentration. Lane 3 is the DNA marker (M). Left panel, a native gel at pH 5.5. Right panel, a denatured gel (10% PAGE, 7 M urea). (DOC) [file pone.0039271.s001.doc]

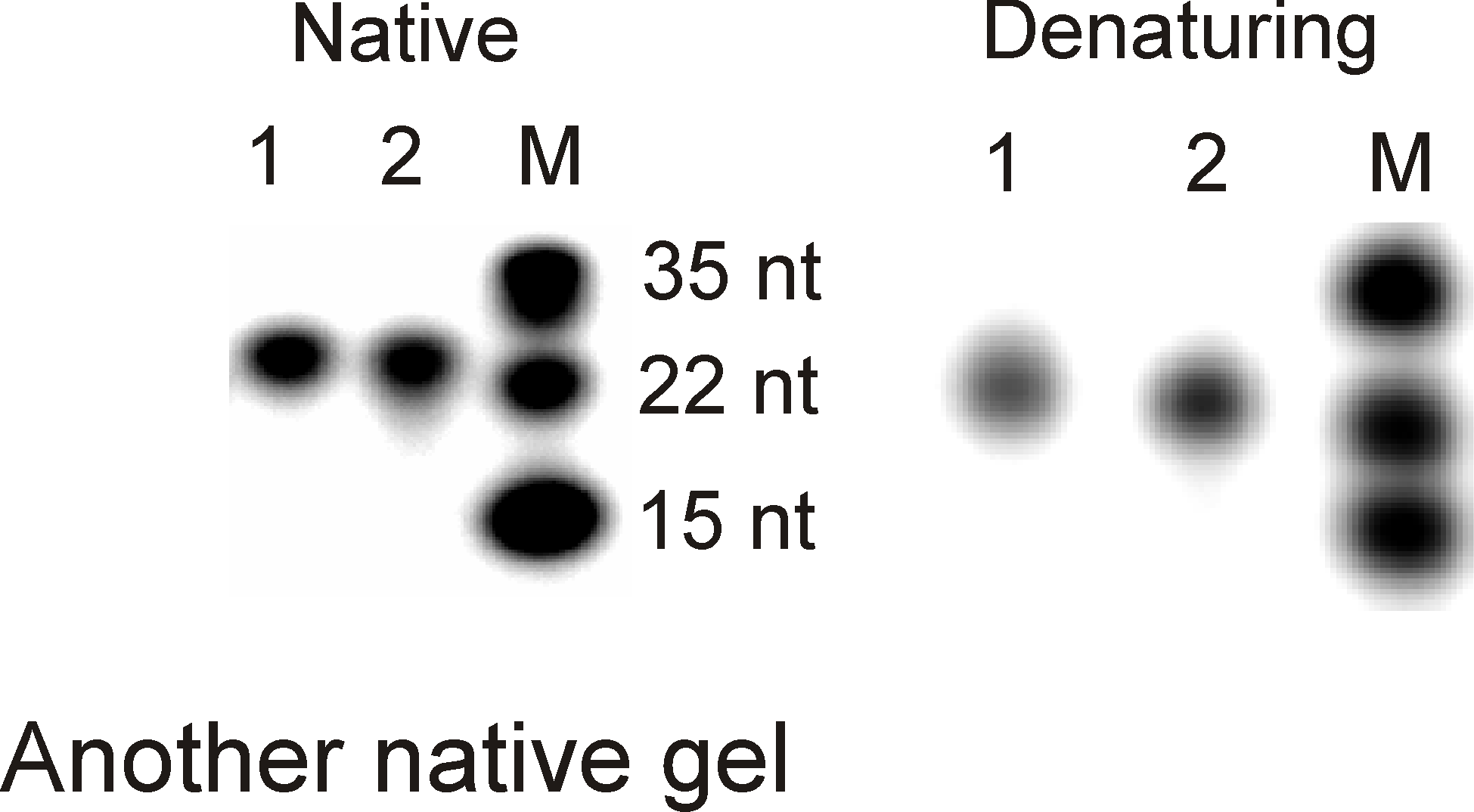


**Figure S1.** Electrophoretic mobility shift assays (EMSA) of the ILPR-I3. EMSA of the ILPR-S3 (scrambled DNA, lane 1) and the ILPR-I3 (lane 2) at 1 μM strand concentration. Lane 3 is the DNA marker (M). Left panel, a native gel at pH 5.5. Right panel, a denatured gel (10% PAGE, 7M urea).
