# Supplementary material for: Intramolecular Folding in Human ILPR Fragment with Three C-Rich Repeats
Source: PLoS One. 2012 Jun 25;7(6):e39271. doi: 10.1371/journal.pone.0039271 (PMC3382603; doi:10.1371/journal.pone.0039271)
Supplement: Figure S2 — CD spectra of sequences described in Table 1. CD spectra of the mutants with mutation sites in each of the three C4 tracts in the ILPR-I3 are plotted in A), B), and C), respectively. The spectra of ILPR-I3 (red) and the scrambled sequence (black) are also included in each Figure for direct comparison. These CD experiments were performed at 5 µM oligonucleotide concentration in a 10 mM sodium phosphate buffer (pH 5.5) with 100 mM KCl at 23°C. (DOC) [file pone.0039271.s002.doc]

**D**

**
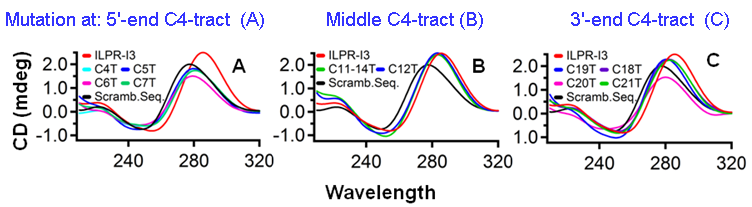
**

**Figure S2.** CD spectra of sequences described in Table 1. CD spectra of the mutants with mutation sites in each of the three C4 tracts in the ILPR-I3 are plotted in A), B), and C), respectively. The spectra of ILPR-I3 (red) and the scrambled sequence (black) are also included in each figure for direct comparison. These CD experiments were performed at 5 µM oligonucleotide concentration in a 10 mM sodium phosphate buffer (pH 5.5) with 100 mM KCl at 23 °C.
