# Supplementary material for: Intramolecular Folding in Human ILPR Fragment with Three C-Rich Repeats
Source: PLoS One. 2012 Jun 25;7(6):e39271. doi: 10.1371/journal.pone.0039271 (PMC3382603; doi:10.1371/journal.pone.0039271)
Supplement: Figure S3 — A typical force-extension (F-X) curve obtained from the mechanical unfolding of the secondary structure in the ILPR-I3 (5'-TGT CCCC ACA CCCC TGT CCCC ACA) at pH 5.5. The unfolding event (∼5 nm) is highlighted by a dashed green circle. Black curve is the WLC fitting of the relaxing curve. Inset is the schematic of the laser tweezers experiment. (DOC) [file pone.0039271.s003.doc]

**
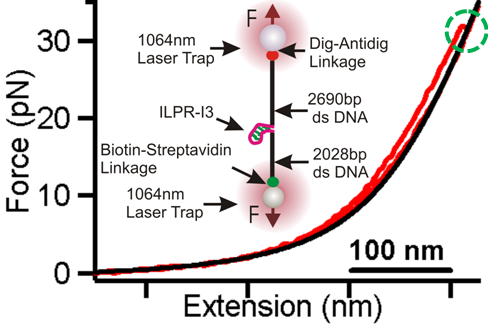
**

**Figure S3.** A typical force-extension (F-X) curve obtained from the mechanical unfolding of the secondary structure in the ILPR-I3 (5'-TGT CCCC ACA CCCC TGT CCCC ACA) at pH 5.5. The unfolding event (~5 nm) is highlighted by a dashed green circle. Black curve is the WLC fitting of the relaxing curve. Inset is the schematic of the laser tweezers experiment.
