# Supplementary material for: Intramolecular Folding in Human ILPR Fragment with Three C-Rich Repeats
Source: PLoS One. 2012 Jun 25;7(6):e39271. doi: 10.1371/journal.pone.0039271 (PMC3382603; doi:10.1371/journal.pone.0039271)
Supplement: Figure S4 — Four possible structures that employ C:CH+ pair stacking in the ILPR-I3 sequence. The three C4 tracts are shown in blue and other regions are shown in red for structures in (A)–(D). The unfolding direction for each structure is shown by black arrows labeled with “F”. Notice the structures with free C4 tracts at the 5'-end yield ΔL values identical to those of B and D, and therefore, they are not shown here. The change in contour length for each structure, ΔL, was calculated (see Table S1 for ΔL values) using the equation, ΔL = N ×L single nucleotide – x (S1), where N is the number of nucleotides involved in the secondary structure, L single nucleotide is the contour length for each nucleotide, and x is the effective end-to-end distance for the folded structure. The x remains the same for the structures A and B, as well as the structures C and D for the mechanical unfolding experiments. To calculate ΔL, the contour length for single nucleotide, L single nucleotide = 0.43 nm, was used as reported (References S1 1–3). The x for structures C&D is 1.5 nm, which is the average inter-phosphate distance obtained from the literature (References S1 4–5). The x for structures A&B was estimated as the hypotenuse (see the green triangles in the top panel) to the rise of the four stacking C:CH+ pairs (the opposite side) and the inter-phosphate distance between the two C4 strands (1.5 nm, the adjacent side). Since the C:CH+ stacking resembles double stranded DNA (dsDNA), we set the lower limit of the rise per C:CH+ as 0.34 nm (single base pair rise in dsDNA) (References S1 6). The upper limit of the rise per C:CH+ is set at 0.66 nm, which is the average rise between the two intercalative C:CH+ stacking pairs determined from the known i-motif structures (PDB Codes; 1YBL, 1G22, 1EL2 and 1CNO) (References S1 7–10). This calculation yielded the rise of the four C:CH+ pairing between 1.0 and 2.0 nm (shown in the left triangle) for structures A and B. Based on this, the x was calculated [file pone.0039271.s004.doc]

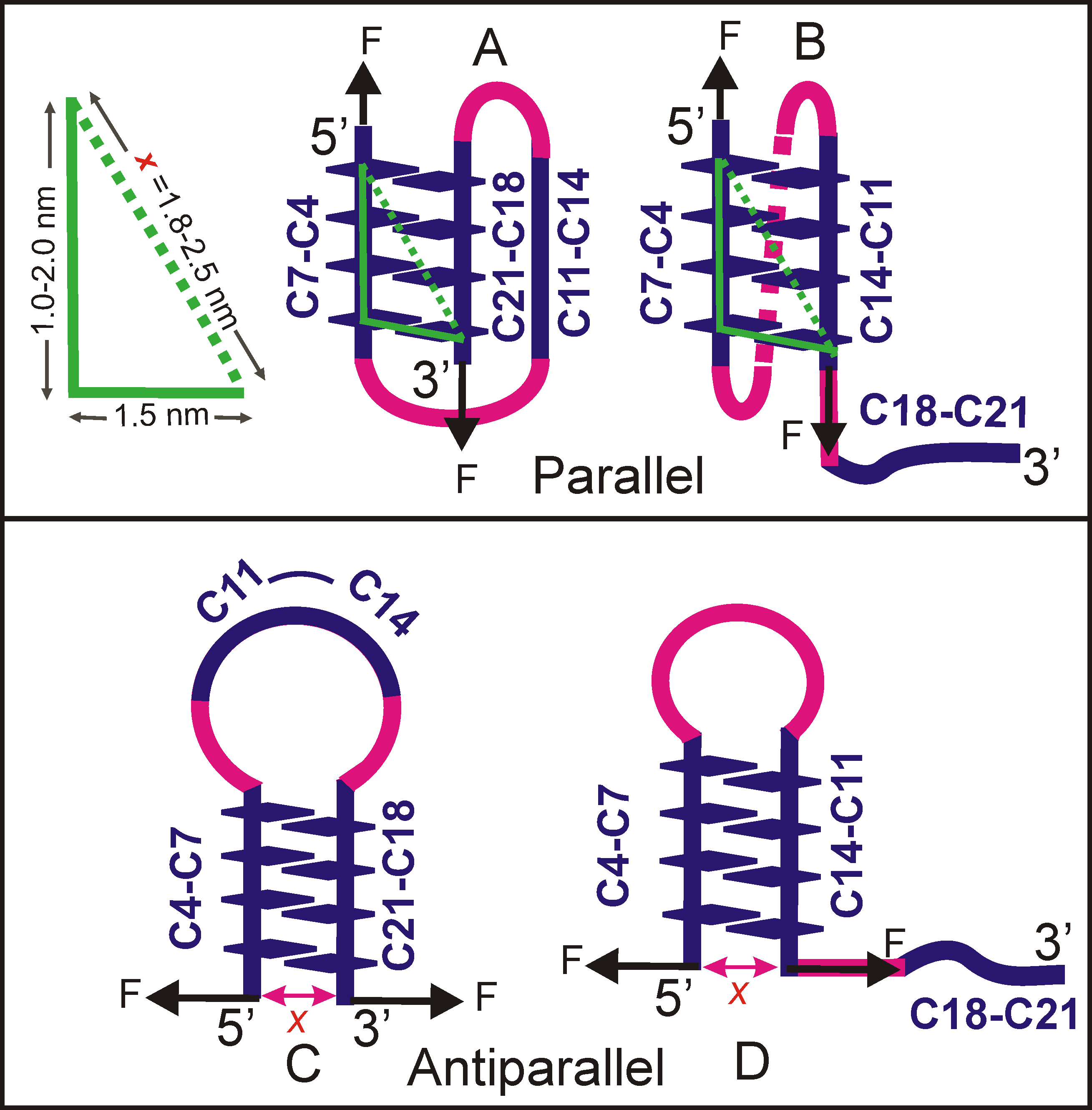


**Figure S4**. Four possible structures that employ C:CH^+^ pair stacking in the ILPR-I3 sequence. The three C4 tracts are shown in blue and other regions are shown in red for structures in (A)-(D). The unfolding direction for each structure is shown by black arrows labeled with “F”. Notice the structures with free C4 tracts at the 5'-end yield ∆*L* values identical to those of B and D, and therefore, they are not shown here.

The change in contour length for each structure, ∆*L*, was calculated (see Table S1 for ∆*L* values) using the equation,

∆*L = N* ×*L*_single nucleotide_ – *x* (S1),

where *N* is the number of nucleotides involved in the secondary structure, *L*_single nucleotide_ is the contour length for each nucleotide, and *x* is the effective end-to-end distance for the folded structure. The *x* remains the same for the structures A and B, as well as the structures C and D for the mechanical unfolding experiments. To calculate ∆*L*, the contour length for single nucleotide, *L*_single nucleotide_= 0.43 nm, was used as reported .

The *x* for structures C&D is 1.5 nm, which is the average inter-phosphate distance obtained from the literature . The *x* for structures A&B was estimated as the hypotenuse (see the green triangles in the top panel) to the rise of the four stacking C:CH^+^ pairs (the opposite side) and the inter-phosphate distance between the two C4 strands (1.5 nm, the adjacent side). Since the C:CH^+^ stacking resembles double stranded DNA (dsDNA), we set the lower limit of the rise per C:CH^+^ as 0.34 nm (single base pair rise in dsDNA) . The upper limit of the rise per C:CH^+^ is set at 0.66 nm, which is the average rise between the two intercalative C:CH^+^ stacking pairs determined from the known i-motif structures (PDB Codes; 1YBL, 1G22, 1EL2 and 1CNO) . This calculation yielded the rise of the four C:CH^+^ pairing between 1.0 and 2.0 nm (shown in the left triangle) for structures A and B. Based on this, the *x* was calculated as 1.8-2.5 nm for these two structures. Using eqn S1, these values yielded ∆*L* of 5.3-5.9 nm and 2.3-2.9 nm for structures A and B, respectively (summarized in the Table S1).

As shown in Table S1, the observed ∆*L* (5.0 ± 0.1 nm at pH 5.5 and 5.2 ± 0.4 nm at pH 7.0) matched with the expected range of ∆*L* (5.3-5.9 nm) for structure A only.
