# Supplementary material for: Intramolecular Folding in Human ILPR Fragment with Three C-Rich Repeats
Source: PLoS One. 2012 Jun 25;7(6):e39271. doi: 10.1371/journal.pone.0039271 (PMC3382603; doi:10.1371/journal.pone.0039271)
Supplement: Figure S5 — Intensity scan for ILPR-I3 bands (the green trace to the left of the gel) and the fold protection for ILPR-I4 (I4, black) and ILPR-I3 (I3, green) for Br2 footprinting in a 10 mM sodium phosphate buffer at pH 5.5 with 100 mM KCl. Note that this gel is identical with that in Figure 5B (see Materials and Methods for fold protection calculation). Samples in different lanes are labeled according to Figure 5B. The cytosines in ACA sections are indicated by blue arrows in the intensity scan and blue bars in the fold protection graphs. The C4 tracts in the gel are highlighted with corresponding sequences. Error bars represent the standard deviations calculated from three independent experiments. (DOC) [file pone.0039271.s005.doc]

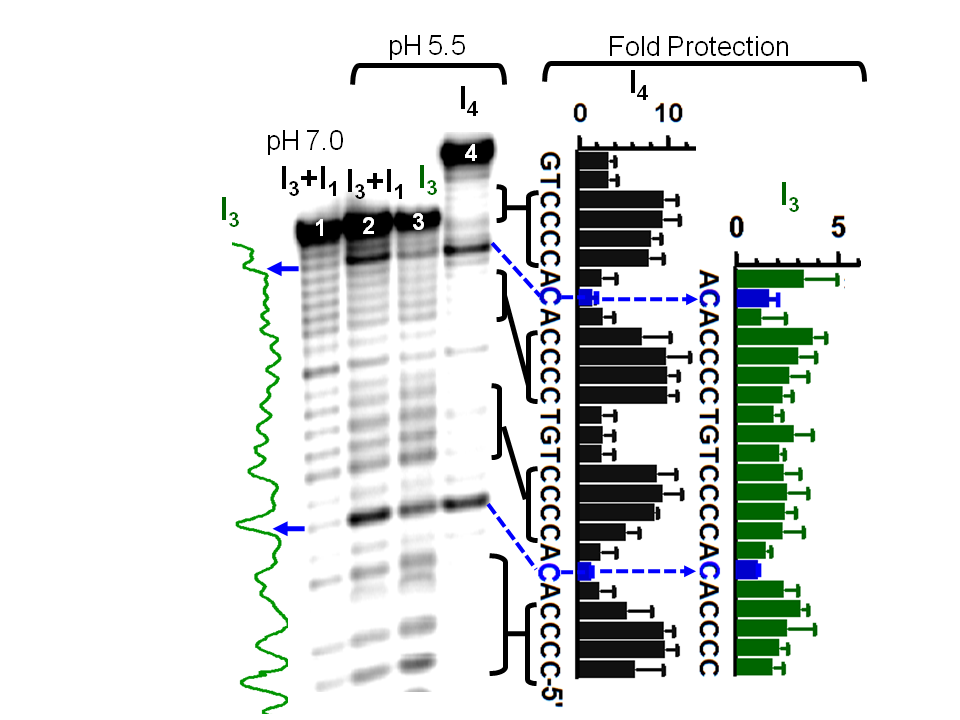


**Figure S5.** Intensity scan for ILPR-I3 bands (the green trace to the left of the gel) and the fold protection for ILPR-I4 (I_4_, black) and ILPR-I3 (I_3_, green) for Br_2_ footprinting in a 10 mM sodium phosphate buffer at pH 5.5 with 100 mM KCl. Note that this gel is identical with that in Figure 5B (see Materials and Methods for fold protection calculation). Samples in different lanes are labeled according to Figure 5B. The cytosines in ACA sections are indicated by blue arrows in the intensity scan and blue bars in the fold protection graphs. The C4 tracts in the gel are highlighted with corresponding sequences. Error bars represent the standard deviations calculated from three independent experiments.
