# Supplementary material for: Intramolecular Folding in Human ILPR Fragment with Three C-Rich Repeats
Source: PLoS One. 2012 Jun 25;7(6):e39271. doi: 10.1371/journal.pone.0039271 (PMC3382603; doi:10.1371/journal.pone.0039271)
Supplement: Figure S6 — Calculation of the unfolding rate constant (k unfold) at 0 pN for the intramolecular i-motif (“ILPR-I4”, calculation based on published data (References S1 References S1 11)) and the 45 pN population in the ILPR-I3/ILPR-I1 mixture (“ILPR-I3+ILPR-I1”) from the plot of ln[r ln(1/N)] versus rupture force. We used the equation S2 to estimate the k unfold, (References S1 12) (S2) where r is the loading rate (5.5 pN/s), N (F,r) is the fraction of folded molecules at force F and loading rate r, and is the distance from the folded state to the transition state along the unfolding coordinate. k unfold is obtained from the linear fit (solid black lines) in the graph. This calculation yielded k unfold of 3.7×10−3 s−1 and 7.2×10−5 s−1, respectively, for the intramolecular ILPR i-motif and the 45 pN population in the ILPR-I3/ILPR-I1 mixture. Notice a randomized deconvolution of the two populations (24 and 45 pN) in the ILPR-I3/ILPR-I1 mixture (Figure 5C, black histogram) was used (References S1 11). (DOC) [file pone.0039271.s006.doc]

**
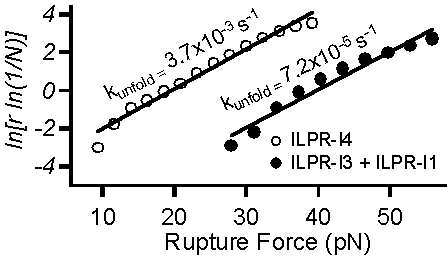
**

**Figure S6.** Calculation of the unfolding rate constant (*k*_unfold_) at 0 pN for the intramolecular i-motif (“ILPR-I4”, calculation based on published data ) and the 45 pN population in the ILPR-I3/ILPR-I1 mixture (“ILPR-I3+ILPR-I1”) from the plot of ln[*r* ln*(1/N)*] versus rupture force. We used the equation S2 to estimate the *k*_unfold_,

$\ln[r\ln[1/N(F,r)]]=\ln[k_{unfold}/(X^{\neq}_{f\to u}/k_{B}T)]+(X^{\neq}_{f\to u}/k_{B}T)F$ (S2)

where *r* is the loading rate (5.5 pN/s), *N (F,r)* is the fraction of folded molecules at force *F* and loading rate *r*, and $X^{\neq}_{f\to u}$ is the distance from the folded state to the transition state along the unfolding coordinate. *k*_unfold_ is obtained from the linear fit (solid black lines) in the graph. This calculation yielded *k*_unfold_ of 3.7×10^-3^ s^-1^ and 7.2×10^-5^ s^-1^, respectively, for the intramolecular ILPR i-motif and the 45 pN population in the ILPR-I3/ILPR-I1 mixture. Notice a randomized deconvolution of the two populations (24 and 45 pN) in the ILPR-I3/ILPR-I1 mixture (Figure 5C, black histogram) was used .
