# Supplementary material for: Intramolecular Folding in Human ILPR Fragment with Three C-Rich Repeats
Source: PLoS One. 2012 Jun 25;7(6):e39271. doi: 10.1371/journal.pone.0039271 (PMC3382603; doi:10.1371/journal.pone.0039271)
Supplement: Table S1 — Calculation of contour length change (ΔL) of the four possible candidates shown in Figure S4. (DOC) [file pone.0039271.s007.doc]

**Table S1. Calculation of contour length change (∆*L*) of the four possible candidates shown in Figure S4**

| **Structures** | **# nucleotides involved (*N*)** | **End to end distance (*x,* nm)** | **Expected change in contour length (∆*L,* nm)** | **Observed (∆*L*, nm)** |
| --- | --- | --- | --- | --- |
| **Fig S4A** | 18 nts | 1.8-2.5 | 5.9-5.3 | 5.0 ± 0.1 (pH 5.5)  5.2 ± 0.4 (pH 7.0) |
| **Fig S4B** | 11 nts | 1.8-2.5 | 2.9-2.3 |
| **Fig S4C** | 18 nts | 1.5 | 6.2 |
| **Fig S4D** | 11 nts | 1.5 | 3.2 |
