# Supplementary material for: Intramolecular Folding in Human ILPR Fragment with Three C-Rich Repeats
Source: PLoS One. 2012 Jun 25;7(6):e39271. doi: 10.1371/journal.pone.0039271 (PMC3382603; doi:10.1371/journal.pone.0039271)
Supplement: References S1 — Supporting Information References. (DOC) [file pone.0039271.s008.doc]

**SUPPORTING INFORMATION REFERENCES:**

1. Tinland B, Pluen A, Sturm J, Weill G (1997) Persistence length of single-stranded DNA. Macromolecules 30: 5763-5765.

2. Record MTJ, Anderson CF, Lohman TM (1978) Thermodynamic analysis of ion effects on the binding and conformational equilibria of proteins and nucleic acids: the roles of ion association or release, screening, and ion effects on water activity. Quart Rev Biophys 11: 103-178.

3. Laurence TA, Kong X, Jager M, Weiss S (2005) Probing structural heterogeneities and fluctuations of nucleic acids and denatured proteins. Proc Nat Acad Sci USA 102: 17348-17353.

4. Mills M, Lacroix L, Arimondo PB, Leroy JL, Francois JC, et al. (2002) Unusual DNA conformations: Implications for Telomeres. Curr Med Chem - Anti-Cancer Agents 2: 627-644.

5. Canalia M, Leroy JL (2005) Structure, internal motions and association-dissociation kinetics of the i-motif dimer of d(5mCCTCACTCC). Nucleic Acids Res 33: 5471-5481.

6. Sinden RR (1995) DNA Structure and Function. California: Academic Press. 165 p.

7. Esmaili N, Leroy JL (2005) i-motif solution structure and dynamics of the d(AACCCC) and d(CCCCAA) tetrahymena telomeric repeats. Nucleic Acids Res 33: 213-224.

8. Nonin-Lecomte S, Leroy JL (2001) Structure of a C-rich strand fragment of the human centromeric satellite III: A pH-dependent interaction topology. J Mol Biol 309: 491-506.

9. Phan AT, Gueron M, Leroy JL (2000) The solution structure and internal motions of a fragment of the cytidine-rich strand of the human telomere. J Mol Biol 299: 123-144.

10. Weil J, Min T, Yang C, Wang S, Sutherland C, et al. (1999) Stabilization of the i-motif by intramolecular adenine-adenine-thymine base triple in the structure of d(ACCCT). Acta Crystallogr, SectD 55: 422-429.

11. Dhakal S, Schonhoft JD, Koirala D, Yu Z, Basu S, et al. (2010) Coexistence of an ILPR i-motif and a partially folded structure with comparable mechanical stability revealed at the single-molecule level. J Am Chem Soc 132: 8991–8997.

12. Li PTX, Collin D, Smith SB, Bustamante C, Tinoco IJ (2006) Probing the mechanical folding kinetics of TAR RNA by hopping, force-jump, and force-ramp methods. Biophys J 90: 250–260.
